# Salt-inducible kinase 3, *SIK3*, is a new gene associated with hearing

**DOI:** 10.1093/hmg/ddu346

**Published:** 2014-07-24

**Authors:** Lisa E. Wolber, Giorgia Girotto, Annalisa Buniello, Dragana Vuckovic, Nicola Pirastu, Beatriz Lorente-Cánovas, Igor Rudan, Caroline Hayward, Ozren Polasek, Marina Ciullo, Massimo Mangino, Claire Steves, Maria Pina Concas, Massilimiliano Cocca, Tim D. Spector, Paolo Gasparini, Karen P. Steel, Frances M.K. Williams

**Affiliations:** 1 Department of Twin Research and Genetic Epidemiology, King's College London, London SE1 7EH, UK,; 2 Medical Genetics, Department of Reproductive Sciences, Development and Public Health, IRCCS-Burlo Garofolo Children Hospital, University of Trieste, Trieste 34100, Italy,; 3 Wolfson Centre for Age-Related Diseases, King's College London, London SE1 1UL, UK,; 4 Centre for Population Health Sciences, University of Edinburgh, Teviot Place, Edinburgh EH8 9AG, UK,; 5 MRC Human Genetics Unit, Institute of Genetics and Molecular Medicine, University of Edinburgh, Western General Hospital, EdinburghEH4 2XU, UK,; 6 Department of Public Health, Faculty of Medicine, University of Split, Split 21000, Croatia,; 7 Institute of Genetics and Biophysics “A. Buzzati-Traverso” CNR, Naples 80131, Italy and; 8 Institute of Population Genetics, National Research Council of Italy, Sassari, Italy

## Abstract

Hearing function is known to be heritable, but few significant and reproducible associations of genetic variants have been identified to date in the adult population. In this study, genome-wide association results of hearing function from the G-EAR consortium and TwinsUK were used for meta-analysis. Hearing ability in eight population samples of Northern and Southern European ancestry (*n* = 4591) and the Silk Road (*n* = 348) was measured using pure-tone audiometry and summarized using principal component (PC) analysis. Genome-wide association analyses for PC1–3 were conducted separately in each sample assuming an additive model adjusted for age, sex and relatedness of subjects. Meta-analysis was performed using 2.3 million single-nucleotide polymorphisms (SNPs) tested against each of the three PCs of hearing ability in 4939 individuals. A single SNP lying in intron 6 of the salt-inducible kinase 3 (*SIK3*) gene was found to be associated with hearing PC2 (*P* = 3.7×10^−8^) and further supported by whole-genome sequence in a subset. To determine the relevance of this gene in the ear, expression of the *Sik3* protein was studied in mouse cochlea of different ages. *Sik3* was expressed in murine hair cells during early development and in cells of the spiral ganglion during early development and adulthood. Our results suggest a developmental role of Sik3 in hearing and may be required for the maintenance of adult auditory function.

## INTRODUCTION

The human ear is one of the most delicate sensory organs of the human body. The cascade of activity resulting in hearing ability transduces the mechanical energy of sound into electrical signals. The inner ear includes the spiral-shaped cochlea containing the organ of Corti, the sensory structure that detects sound. Here, stereocilia on the apical surface of sensory hair cells sense movement of the surrounding fluids and open ion channels upon stereocilia deflection. The resulting depolarization of the hair cells initiates synaptic activity of auditory neurones. Inner hair cells are innervated via mostly unbranched and myelinated type I spiral ganglion neurones, whereas the rather thinner and unmyelinated type II spiral ganglion neurones innervate outer hair cells (1,2). Failure of any of the components may result in impaired hearing ability or hearing loss (HL).

Twin studies suggest a role for genetic factors in hearing ability, with estimates of heritability of up to 70–75% (3–5). Several environmental risk factors for diminished hearing ability have been identified, including noise exposure (6,7), cardiovascular disease (8), ototoxic medication (9) and smoking (7). Genetic risk factors, for the most part, remain to be accurately determined. Candidate gene studies have supported associations of proteins involved in reactive oxygen species removal including N-acetyl-transferase 2 (*NAT2*), glutathione S-transferase theta 1 (*GSST1*) and glutathione S-transferase Mu 1 (*GSTM1*) with presbycusis (10,11). Various studies have tried to elucidate the genetic background of adult hearing function and age-related HL in genome-wide linkage (12–14) and association studies (14–17). In these studies, several genes involved in age-related HL have been identified, including metabotropic glutamate receptor 7 (*GRM7*) and IQ motif containing GTPase activating protein 2 (*IQGAP2*). Furthermore, a meta-analysis of hearing function revealed associations with a series of genes including metabotropic glutamate receptor 8 (*GRM8*) and protein tyrosine phosphatase receptor type *D* (*PTPRD*) (17). However, confirmation of findings or study of expression in model organisms has been reported for only a few studies (15,17). Adult hearing is likely to involve many common genetic variants each having low effect sizes. In common with many complex traits, only a small proportion of the heritability is explained by the genetic variants identified so far.

In order to optimize power by maximizing sample size, we performed a GWAS meta-analysis of eight European samples and one Silk Road population sample (18), having hearing ability measured by pure-tone audiometry and summarized by principal components (PCs). Genetic variants found associated with hearing ability were examined further for expression of the gene product in mouse cochlear structures.

## RESULTS

### Subjects

The characteristics of the 4939 individuals are summarized by sample in Table 1. Age for the different study samples ranged from 18 to 98 years. There was a preponderance of female participants (55%–100%) across the groups. Samples were included from TwinsUK and the G-EAR consortium; the latter were originally recruited to study hearing function in isolated populations (19,20) and were collected from several isolated villages, as described previously (20). Samples from the TwinsUK collection included same-sex twin pairs as part of a project to study ageing. Principal component analysis was used to summarize the pure-tone audiogram data (14). In TwinsUK, the first three PCs of the pure-tone audiogram accounted for 87% of the variance in the data. PC1 represented a measure of the horizontal threshold shift in the audiogram and, in TwinsUK, was highly correlated with the pure-tone average calculated over all frequencies (*r* = −0.98). PC2 represented the downward slope of the audiogram reflecting worse thresholds at higher frequencies, and PC3 gave a measure of audiogram concavity (how much the audiogram deviates from a straight line). While PC1 values were negatively correlated with raised pure-tone thresholds, high-frequency HL (resulting in a downward slope from the lower to the higher frequencies of the audiogram) was positively correlated with PC2.

**Table 1.** Population characteristics for subjects from the G-EAR consortium and TwinsUK

Population	Country	Sample size	Gender (% females)	Age range (years)	Mean age (± SD)
Carlantino	Italy	280	56.87	18–89	53.29 (18.1)
Friuli Venezia Giulia	Italy	1097	60.19	18–89	51.47 (16.3)
Korcula	Croatia	804	63.30	18–98	56.3 (13.7)
Split	Croatia	497	56.00	18–79	49.0 (14.6)
Cilento	Italy	421	56.67	18–91	56.3 (17.6)
Talana	Italy	470	59.00	18–92	50.82 (18.5)
Silk Road	Azerbijan	348	54.60	18–82	41.59 (15.5)
	Giorgia				
	Kazakistan				
	Tajikistan				
	Uzbekistan				
Twins UK	UK	1022	100	29–86	61.06 (9.1)
Total	–	4939	–	18–98	–

Characteristics of the eight different populations by country of origin, sample size (*n*), gender and age range in years as well as mean age and standard deviation (SD) from mean age.

### Meta-analysis

**Figure 1.**
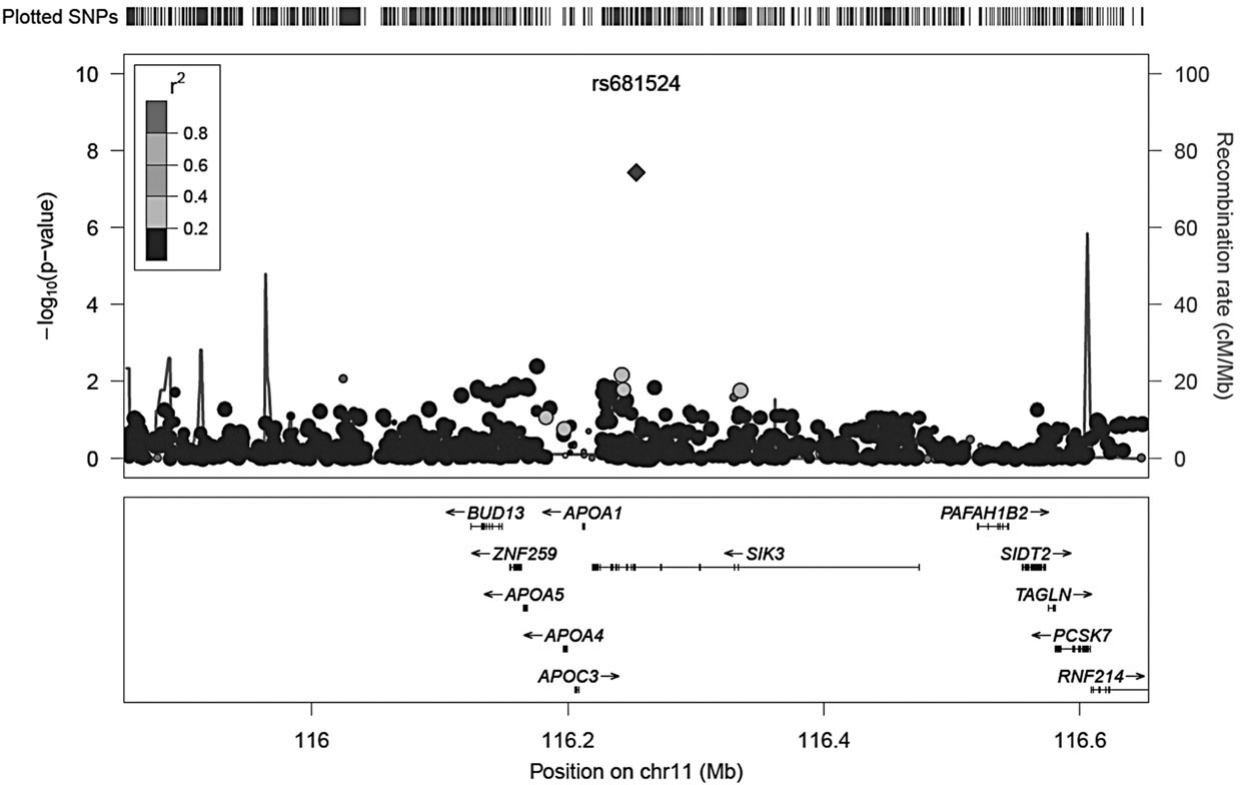
Locus zoom plot of the associated genetic markers on chromosome 11q23.3. The locus zoom depicts the location of genetic markers versus their significance of association with PC2. Significance of association is measured as the negative logarithm of the *P*-value. Genes located in the area 400 kb up-and downstream of rs681524 are displayed below the *x*-axis. The grey shading of each genetic marker indicates its correlation with the reference SNP rs681524. A legend for the correlation is shown in the upper left corner of the locus zoom. Recombination rate is highlighted as peaked lines.

**Figure 2.**
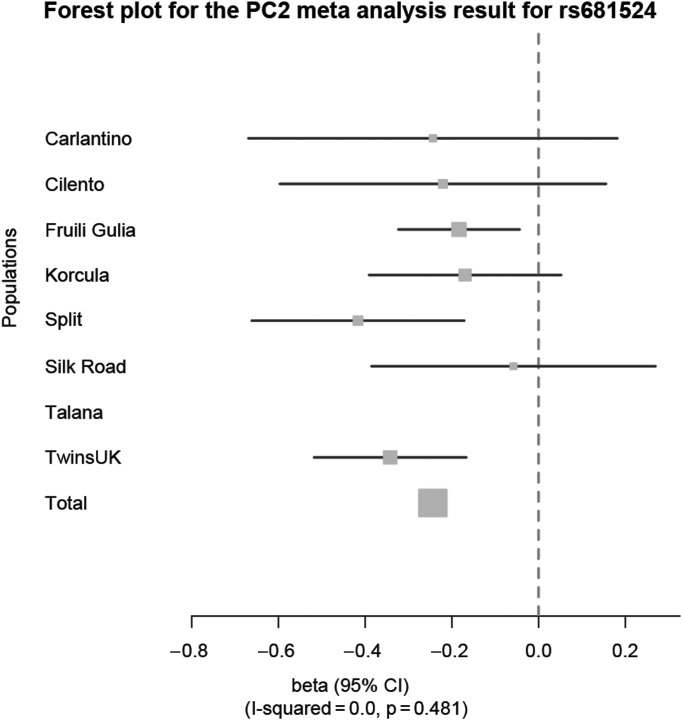
Forest plot for the PC2 meta-analysis result for rs681524 in *SIK3.* The forest plot shows the effect sizes (beta with 95% CI) at rs681524 on PC2 for each population in comparison with a combined meta-analysis effect (total). A consistent negative effect for the minor C allele at rs681524 for all European populations could be determined. The percentage of variation owing to heterogeneity across populations was not significant (*I*^2^ = 0.0, *P* = 0.481). Corresponding data are listed in Table 5.

Single-nucleotide polymorphisms (SNPs) most significantly associated in GWAS meta-analysis of PC1, PC2 and PC3 are shown in Tables 2, 3 and 4, respectively. After quality control (described in Supplementary Material, Table S1) and imputation, >2.3 million SNPs were examined. The complete set of GWAS meta-analyses results can be accessed under following link: http://www.twinsuk.ac.uk/wp-content/uploads/TUK_G-EAR_GWAS_hearing.zip, last accessed on 7 July 2014. There was a single genome-wide significant SNP (*P* < 5 × 10^−8^) on chromosome 11 associated with PC2, the PC representing the slope for higher frequencies of the audiogram (Fig. 1, locus zoom of SNP ±400 kb). A forest plot of the results for this SNP rs681524 is shown in Figure 2 with corresponding data in Table 5. This plot shows the estimated effect sizes [beta and 95% confidence interval (CI)] of the C allele at rs681524 for different samples and a combined meta-analysis effect (total beta = −0.24). The SNP was genotyped in TwinsUK and imputed in the other samples but was not available in the sample from Talana, so meta-analysis was performed on seven of the eight groups (*n* = 4322). The forest plot shows a consistent direction of effect of this allele across all seven samples, which is nominally significant in three of these (Split, Talana and TwinsUK, Table 5). We further calculated the percentage of total variation across the seven populations owing to heterogeneity in form of *I*^2^ (21). There was no observed heterogeneity (*I*^2^ = 0.0, *P* = 0.481) for the association with rs681524. To control for differences in allele frequency between the different samples, data from the human genome diversity project (22) were used. No significant differences could be detected for rs681524 in the samples studied here. The locus zoom in Figure 1 shows little in the way of supporting SNPs associated with the hearing phenotype. Further evidence that this finding was not a false-positive was sought: visual inspection of the cluster plots of rs681524 in TwinsUK revealed clear separation of the alleles (Supplementary Material, Table S2). Exploration of the linkage disequilibrium (LD) pattern surrounding SNP rs681524 (in a 1-Mb window surrounding rs681524) was made using data from the 1000 Genomes Project (23) using the SNP Annotation and Proxy Search tool (24) and the TwinsUK cohort whole-genome sequencing sample as part of the UK10K project. The data from the 1000 Genomes project confirmed that there were only 5 SNPs in moderate LD (*r*^2^ = 0.8–0.6) with rs681524, 4 of which were located in intronic regions of the *SIK3* gene and 1 SNP in an intron of the *PCSK7* gene. LD information calculated from the UK10K data revealed even lower LD in the proximity of rs681524 (*r*^2^ < 0.28 in 400 neighbouring SNPs in a window of 1 Mb surrounding rs681524).

**Table 2.** Meta-analysis results for PC1

SNP	Allele 1	Allele 2	Z-score	*P*-value	direction	Chr	Position	Gene	Feature	Left gene	Right gene
rs589636	t	c	−5.40	6.61E-08	?------?	13	76414577	IRG1	intron	BTF3L1	LOC390413
rs588702	t	c	−5.33	9.75E-08	?-------	13	76420926	IRG1	intron	BTF3L1	LOC390413
rs2687481	t	g	−5.32	1.07E-07	-------?	7	125656358	NA	NA	LOC646837	GRM8
rs2521030	c	g	−5.23	1.69E-07	-------?	7	125656552	NA	NA	LOC646837	GRM8
rs614171	a	g	−5.23	1.71E-07	?-------	13	76414753	IRG1	intron	BTF3L1	LOC390413

Five SNPs were suggestive genome-wide significant (*P* < 5 × 10^−7^) associated with PC1. Meta-analysis results for PC1 are further characterized by non-effect allele (allele 1) and effect allele (allele 2), the resulting Z-score and significance of association (*P*-value). The direction of effect (minus or plus) is indicated for each of the eight included populations. If an SNP did not pass QC criteria for a certain SNP, this is indicated by a question mark (?) in the direction column. Mapping information for each SNP is specified by chromosome (chr), base-pair position (position), genes at this locus (gene) and surrounding the locus (left and right gene) as well as feature of the SNP position within a gene (feature).

**Table 3.** Meta-analysis results for PC2

SNP	Allele 1	Allele 2	Z-score	*P*-value	Direction	Chr	Position	Gene	Feature	Left gene	Right gene
rs681524	t	c	−5.505	3.69E-08	?-------	11	116253524	SIK3	intron	APOA1	LOC100129905
rs1393902	a	g	5.119	3.07E-07	++++++++	8	68584119	CPA6	intron	ARFGEF1	LOC100132812
rs1503369	t	c	5.116	3.12E-07	++++++++	8	68584552	CPA6	intron	ARFGEF1	LOC100132812
rs1827524	a	g	5.104	3.32E-07	++++++-+	8	68587796	CPA6	intron	ARFGEF1	LOC100132812
rs1393901	t	c	5.100	3.40E-07	++++++-+	8	68587888	CPA6	intron	ARFGEF1	LOC100132812
rs6472312	t	g	−4.994	5.92E-07	------+-	8	68572052	CPA6	intron	ARFGEF1	LOC100132812
rs1503363	a	g	−4.940	7.80E-07	------+-	8	68569258	CPA6	intron	ARFGEF1	LOC100132812

Seven SNPs were suggestive genome-wide significant (*P* < 5 × 10^−7^) associated with PC2. Top associated SNPs for PC2 are further characterized by corresponding non-effect allele (allele 1) and effect allele (allele 2), the resulting Z-score and significance of association (*P*-value). The direction of effect (minus or plus) is indicated for each of the eight included populations. If an SNP did not pass QC criteria for a certain SNP, this is indicated by a question mark (?) in the direction column. Mapping information for each SNP is specified by chromosome (chr), base-pair position (position), genes at this locus (gene) and surrounding the locus (left and right gene) as well as feature of the SNP position within a gene (feature).

**Table 4.** Meta-analysis results for PC3

SNP	Allele 1	Allele 2	Z-score	*P*-value	Direction	Chr	Position	Gene	Feature	Left gene	Right gene
rs6134479	c	g	5.010	5.44E-07	+++++++?	20	12170165	NA	NA	BTBD3	PA2G4P2

One SNP was suggestive genome-wide significant (*P* < 5 × 10^−7^) associated with PC3. The top associated SNP for PC3 is further characterized by corresponding non-effect allele (allele 1) and effect allele (allele 2), the resulting Z-score and significance of association (*P*-value). The direction of effect (minus or plus) is indicated for each of the eight included populations. If an SNP did not pass QC criteria for a certain SNP, this is indicated by a question mark (?) in the direction column. Mapping information for each SNP is specified by chromosome (chr), base-pair position (position), genes at this locus (gene) and surrounding the locus (left and right gene) as well as feature of the SNP position within a gene (feature).

**Table 5.** GWAS and meta-analysis results for rs681524

Population	*n*	MAF	Imputation quality	Beta	SE	*P*-value	Z-Score
Carlantino	280	0.057	0.458	−0.2438	0.2215	0.2710	−1.101
Cilento	419	0.075	0.438	−0.2206	0.1958	0.2599	−1.127
Friuli Venezia Giulia	1097	0.080	0.696	−0.1836	0.0728	0.0117	−2.522
Korcula	794	0.064	0.523	−0.1695	0.1153	0.1415	−1.470
Split	497	0.087	0.598	−0.4166	0.1276	0.0011	−3.265
Silk Road	255	0.039	0.618	−0.0579	0.1705	0.7342	−0.340
Talana	–	–	–	–	–	–	–
TwinsUK	980	0.065	1.00	−0.3423	0.0914	1.8E-04	−3.745
Total	4322	–	–	−0.2439	–	3.69E-08	−5.505

The GWAS results for each population at rs681524 are characterized by the number of subjects with genotyping or imputation data at this SNP (*n*), the direction of effect for the C allele (beta), the MAF of the effect allele, the imputation quality of the SNP, standard error of the direction of effect (SE) and significance of association and Z-score calculated for the meta-analysis. The combined meta-analysis results are listed as Total. A graphical interpretation of this data can be seen in Figure 2. SNP rs681524 did not pass quality control criteria for the population of Talana.

The accuracy of imputation of rs681524 in all samples but from TwinsUK could be assessed in two samples from the G-EAR consortium (Carlantino and Friuli Venezia Giulia), subsets of which had whole-genome sequencing data available in addition to the original imputation (Table 6). Imputation accuracy accessed as genotype concordance at rs681524 ranged from 0.97 to 0.94 (for Carlantino and Friuli Venezia Giulia, respectively). A comparison of genotyping at rs681524 between the original GWAS and the TwinsUK UK10K whole-genome sequence gave a genotype concordance rate of 0.99 (Table 6).

**Table 6.** Imputation accuracy at rs681524 in whole-genome sequencing data

Population	Genotyping/imputation accuracy	
	*n*	Genotype concordance
Carlantino	93	0.97
Friuli Venezia Giulia	222	0.94
TwinsUK	2000	0.99

Imputation quality was assessed in two subsamples from Carlantino and Friuli Venezia Giulia with whole-genome sequencing data available. Imputation accuracy was measured as genotype concordance rate at rs681524. For TwinsUK, genotyping data from the original GWAS sample were compared with whole-genome sequencing data from the TwinsUK UK10K sample.

In addition, 503 females from the TwinsUK UK10K whole-genome sequencing sample had completed a hearing test. A linear regression analysis in this subset confirmed a negative association between PC2 and rs681524 genotype (adjusted for age and twin relatedness) (*P* = 0.010, beta ± SE = −0.33 ± 0.13).

### Association analysis with rs681524 by age groups

As our interest lies in age-related hearing impairment, subgroup analysis of rs681524 by age was performed. Association analyses were conducted for under 40 years, 40 to 60 years and older than 60 years. The protective effect of the C allele of rs681524 was detectable in all three age groups with a mean effect ranging from −0.117 to −0.386 (Table 7). The largest effect could be detected in the youngest samples (>40 years) although this was the smallest sample (Table 7).

**Table 7.** Association of rs681524 with hearing PC2 stratified by age groups

Population	<40 years			40–60 years			>60 years		
	*n*	Beta	SE	*n*	Beta	SE	*n*	Beta	SE
Carlantino	88	−0.448	0.613	87	0.468	0.599	105	−0.412	0.403
Cilento	82	−0.956	0.385	150	−0.675	0.282	188	0.105	0.274
Friuli Venezia Giulia	295	0.124	0.116	424	−0.518	0.170	378	−0.258	0.210
Korcula	103	0.089	0.306	399	0.304	0.183	292	0.349	0.243
Split	138	−0.307	0.257	232	−0.595	0.218	127	−0.263	0.272
Silk Road	115	−0.210	0.424	104	0.253	0.428	36	−0.027	0.691
Talana	NA	NA	NA	NA	NA	NA	NA	NA	NA
TwinsUK	19	−0.993	0.671	372	−0.356	0.162	589	−0.314	0.113
Mean effect	120	−0.386	0.396	253	−0.160	0.292	245	−0.117	0.315

The effect of the C allele at rs681524 on hearing PC2 was analysed in three age groups (>40, 40–60 and >60 years) in all samples separately. For each association, the sample size (*n*), effect size (beta) and standard error of the effect size (SE) are given. A mean effect was calculated for all samples per age group (beta, SE).

### Expression analysis of Sik3

**Figure 3.**
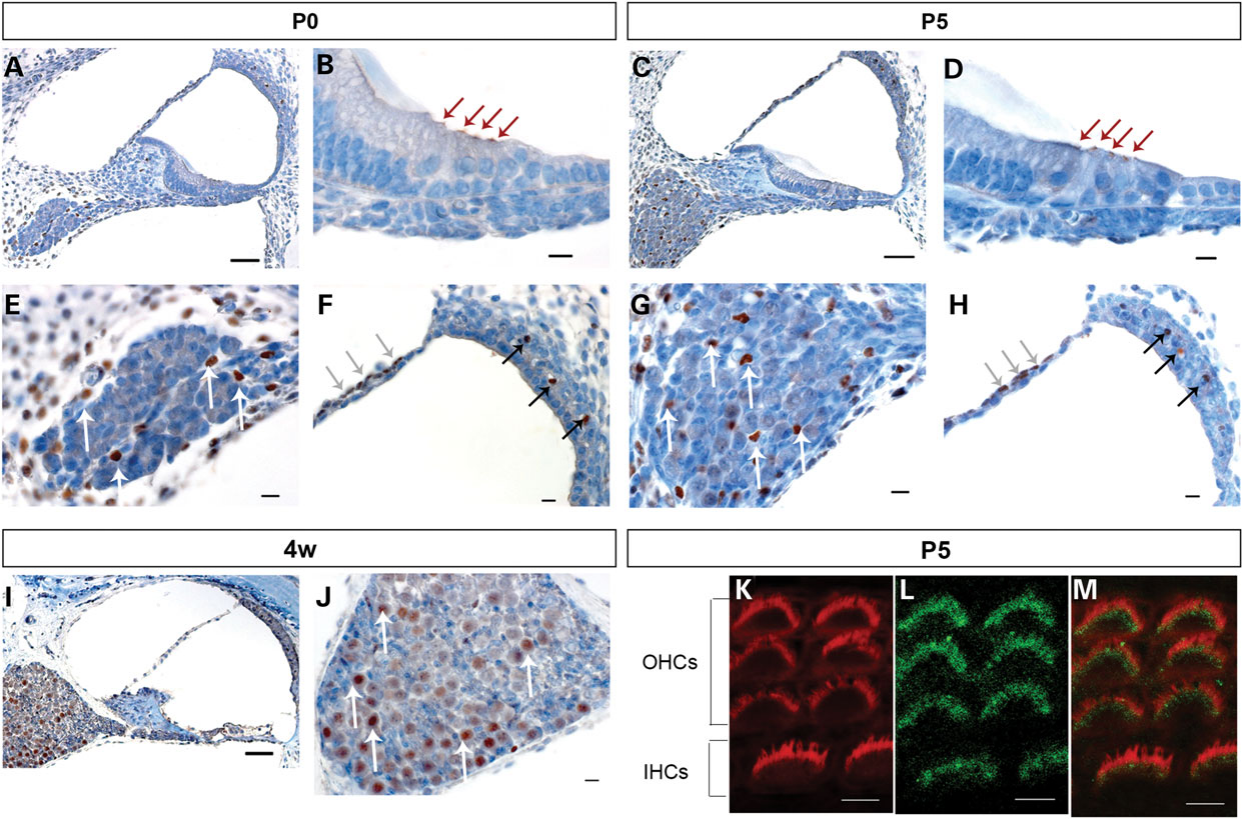
Sik3 is expressed in cochlear structures at different developmental stages. Mouse cochlear sections were stained with primary Sik3 antibody (brown) and counterstained with haematoxylin (blue). **A, B, E** and **F**: at the day of birth (P0), Sik3 expression was detected in the apex of inner and outer hair cells (B, red arrows), in the perilymph-facing layer of the Reissner's membrane (F, grey arrows), near blood vessels of the intermediate layer of the stria vascularis (F, black arrows), as well as in cells of the spiral ganglion (E, white arrows) and cells surrounding the ganglion. **C, D, G** and **H**: at 5 days postnatal (P5), Sik3 expression was found in the hair cells (D, red arrows) and small cells of the spiral ganglion (G, white arrows). **I** and **J**: at 4 weeks postnatal, Sik3 expression remained in the spiral ganglion (J, white arrows), Reissner's membrane and stria vascularis but could not be detected in the apex of the hair cells (I). **K, L** and **M**: confocal imaging of Sik3 expression in the stereocilia at 5 days postnatal (L, green), compared with Phalloidin expression (K, red) showed that Sik3 was expressed in the region around the base of the stereocilia (M, merged image of K and L). IHCs, inner hair cells; OHCs, outer hair cells. Scale bars: A, C and I: 50 µm; B, D, E, F, G, H and J: 10 µm; K, L and M: 5 µm.

Sik3 was highly expressed at all three developmental stages studied in the mouse: at the day of birth as well as 5 days postnatal, expression of Sik3 was limited to the tops of the inner and outer hair cells in the cochlea, non-neuronal cells of the spiral ganglion, cells in the intermediate layer of the stria vascularis and the perilymph-facing layer of the Reissner's membrane (Fig. 3). In the stria vascularis, Sik3 was expressed near blood vessels in the intermediate layer. These cells have previously been described as macrophage-like melanocytes (25). At 4 weeks after birth, Sik3 expression could no longer be detected in the hair cells, indicating that this expression was limited to early developmental stages. Expression in the spiral ganglion and stria vascularis remained even 4 weeks after birth (Fig. 3).

To confirm whether Sik3 expression in the hair cells is limited to the apex of the hair cells or the stereocilia, confocal imaging of 5-day-old whole-mount sections was performed. Sik3 expression was found at the top of hair cells in the region around the base of the stereocilia in both inner and outer hair cells throughout the cochlea, from base to apex (Fig. 3).

### Differential Sik3 expression in the spiral ganglion

**Figure 4.**
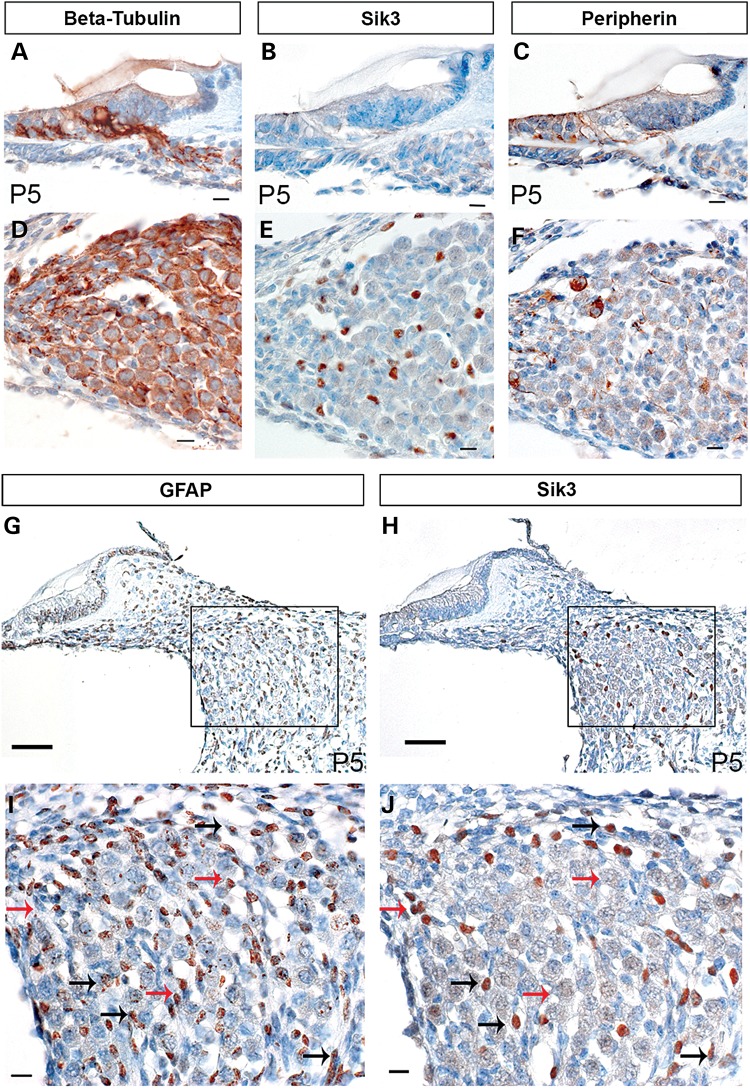
Sik3 is expressed in small cells between the neurones of the spiral ganglion. To identify the cell type expressing Sik3 in the spiral ganglion, Sik3 expression was compared with beta-tubulin **(A** and **D**), Peripherin (**C** and **F**) and GFAP (**G** and **I**) expression in adjacent sections. Beta-tubulin is expressed in both Type 1 and 2 spiral ganglion neurones, whereas peripherin expression is limited to type 1 spiral ganglion neurones. Sik3 expression (**B** and **E**) did not coincide with either beta-tubulin (A and D) or peripherin expression (C and F) but seemed to be expressed in smaller cells interspersed between the neurones. To determine whether Sik3 could be expressed in glial cells, Sik3 expression (**H** and **J**) was compared with GFAP expression (G and I) in adjacent sections. Sik3 and GFAP expression overlapped partially. Sik3 expression coincided with GFAP expression in some cells (black arrows) but was absent in other glial cells (red arrows). All sections were prepared from mice at 5 days postnatal (P5). Expression of each antibody is indicated by a brown signal. Scale bars: A, B, C, D, E, F, I and J: 10 µm; G, H: 50 µm.

We performed a parallel stain of adjacent spiral ganglion sections at 5 days old with markers specific for Type 1 and 2 spiral ganglion neurones and glial cells. Peripherin is expressed in type 2 neurones only (26), whereas β-tubulin Tuj1 is expressed in both type 1 and 2 spiral ganglion neurones (27), and glial fibrillary acidic protein (GFAP) expression is limited to Schwann and satellite cells in the developing spiral ganglion (28). The differential staining pattern of the markers was compared with Sik3 expression in the spiral ganglion, and there was no overlap with either peripherin or beta-tubulin (Fig. 4). These findings suggest that Sik3 is not expressed in the neurones themselves, but in surrounding structures. We went on to compare Sik3 expression with that of GFAP, a marker for glial cells. GFAP was widely expressed in non-neuronal cells of the spiral ganglion, whereas Sik3 expression appeared in more limited numbers of small cells in the spiral ganglion (Fig. 4). Sik3 appears to be expressed in the cell nucleus, whereas GFAP is expressed in the cytoplasm and cytoskeleton of non-neuronal cells. Careful analysis of adjacent sections labelled with Sik3 and GFAP antibodies suggested that they labelled partly overlapping populations of non-neuronal/glial cells, with some cells expressing only Sik3, some only GFAP and other cells labelled for both proteins (Fig. 4). A similar expression profile in the spiral ganglion has been reported for oestrogen receptor-β previously (29).

## DISCUSSION

Here, we report results from the largest GWAS meta-analysis on hearing ability to date, comprising 8 study groups from across Europe, giving a total sample size of 4939 adults, allowing phenotype- and population-specific adjustments to be performed for each GWAS separately. We have identified a single SNP in *SIK3* (*P* = 3.7 × 10^−8^) as associated with hearing.

Concern that the finding of the single associated SNP on chromosome 11 represented a false-positive was addressed in a number of ways. Examination of the region of interest revealed an unusual pattern of LD: 1000 Genomes and UK10K data reflecting genetic variations from 1092 and 2000 genomes, respectively, were found to be consistent with HapMap CEU data, with very low LD across the region. Examination of genotyping cluster plots (Supplementary Material, Fig. S2) in the TwinsUK sample revealed clear separation of genotypes at rs681524. Although this SNP was genotyped in TwinsUK only, imputation quality of rs681524 passed our quality control threshold of 0.3 in the other six population samples (Table 5). Furthermore, imputation accuracy for rs681524 as determined in subsets from Friuli Venezia Giulia and Carlantino with whole genotyping data (*n* = 222 and 93, respectively) ranged between 0.94 and 0.97, respectively. The *SIK3* SNP showed significant genome-wide association with PC2, and the gene was considered a good candidate for hearing ability because of the involvement of other salt-inducible kinases (SIKs) in the inner ear (30). Immunohistochemistry was performed using mouse cochlea in order to localize expression of the protein to a single cell type, rather than to the cochlea in general. Sik3 was found expressed in the apex of the hair cells of murine cochlea during early development and in non-neural cells of the spiral ganglion throughout development and adulthood. Furthermore, it was expressed in macrophage-like melanocytes in the stria vascularis and perilymph-facing cells at the Reissner's membrane. Confocal microscopy confirmed the expression of Sik3 at the apex of the hair cells around the bases of the stereocilia of 5-day-old wild-type mice. Expression of Sik3 in the apex of the hair cells was limited to early stages of development. Mouse pups are born deaf and develop the ability to hear at around Day 12 (31). Our data suggest that Sik3 may be important for hair cell or stereocilia development but is not required for the maintenance of these structures in adulthood. In contrast, Sik3 was consistently expressed in spiral ganglia throughout all developmental stages. The striking expression of Sik3 in the murine cochlea supports a role during development and maintenance of the inner ear.

We compared the expression of *Sik3* with that of molecular markers for Type 1 and Type 2 spiral ganglion neurones and glial cells. Sik3 expression in the spiral ganglion did not overlap with neurones but with smaller, distinct cells interspersed between them. Comparison of Sik3 and GFAP, a marker for glial cells, in adjacent cochlear sections showed Sik3 limited to the nucleus of labelled cells, whereas GFAP was expressed in cytoplasmic structures of non-neuronal cells of the spiral ganglion. Sik3 and GFAP expression overlapped partially in the spiral ganglion. In conclusion, Sik3 is expressed in non-neuronal cells of the spiral ganglion, which we assume to be an undefined subgroup of glial cells.

SIKs belong to the family of cAMP-activated serine threonine kinases (32). Three family members have been described so far (32), and isoforms 1 and 2 have been the main focus of research. Sik1 and 2 show high expression in adrenal glands of mice on a high sodium diet, thus the name “salt-inducible” kinases, whereas Sik3 was reported to be expressed ubiquitously (32). Sik1 has been linked to various processes, including sodium sensing and cardiomyogenesis (33,34). In addition, Sik1 has been shown to be expressed in the inner ear, in the sensory epithelium of the vestibular system, where it is thought to be involved in the formation of hydrops via interaction with phosphodiesterases (30). Both Sik1 and Sik3 control histone deacetylases via phosphorylation and nuclear export (35–37). *Sik3* knockout mice (*Sik3^−/−^*) have a high mortality rate at birth. Surviving pups show skeletal abnormalities, reduced bodyweight and dwarfism, but no details of their hearing ability have been reported yet. *Sik3^−/−^* mice show reduced energy storage, which is associated with hypoglycaemia and hyper-insulin sensitivity. Lipid metabolism disorders in (*Sik3^−/−^*) mice were partially restored after 9-cis-retionic acid supplementation (38). *Sik3* is assumed to regulate cholesterol bile acid homeostasis and lipid storage size and is essential for chondrocyte hypertrophy (36,38). In addition, SIKs have recently been shown to be involved in the formation of regulatory macrophages (39). Salt-inducible kinases were responsible for phosphorylation of the CREB-regulated transcriptional co-activator 3 and could therefore inhibit the formation of regulatory macrophages from proinflammatory macrophages (39). It appears that Sik3 may act as an important regulator in various body systems, and any of the above roles of Sik3 could affect the long-term maintenance and function of the auditory system. From our findings, the Sik3 expression pattern in the mouse inner ear suggests that it is important during development of hair cells but less so for the maintenance of these sensory structures. Furthermore, expression data in mice suggested a maintenance function of Sik3 in cells of the spiral ganglion. Examining hearing function over time in *Sik3* knockout mice will be essential to understand fully the function of *Sik3* in hearing ability and to give more detail about putative mechanistic links.

A subset of the samples used in this GWAS meta-analysis had been used previously in a GWAS meta-analysis of hearing function (17). In the present study, we included two further studies of hearing function, one a population sample situated along the Silk Road (Azerbijan, Giorgia, Kazakistan, Tajikistan, Uzbekistan)(20) and the other a large sample of females from the TwinsUK collection. By increasing the sample size, and therefore statistical power, it was hoped to determine associations with hearing of genome-wide significance. In comparison with the previously performed meta-analysis, the overlap of significant associations was limited. The association of rs681524 has not been reported in the previous study, which is consistent with the finding that this association was strongly supported by the newly added TwinsUK GWAS (Table 5, *P*-value (TwinsUK) = 1.8 × 10^−4^). Furthermore, the previous study reported suggestive significant associations with metabotropic glutamate receptor type 8 (PC1, *P*-value = 3.22 × 10^−7^); the PBX/knotted homeobox 2 gene (PC2, *P*-value = 2.86 × 10^−7^) and phosphatidic acid phosphatase type 2D (PC3, *P*-value = 2.32 × 10^−7^) (17). The association between PC1 and SNPs in the vicinity of *GRM8* was confirmed in the present study (rs2687481, *P* = 1.07 × 10^−7^), whereas the association we report here between PC1 and intronic SNPs in Immunoresponsive 1 homologue (*IRG1*, rs589636, *P* = 6.61 × 10^−8^) has not been described previously. Furthermore, the association of PC2 with carboxypeptidase A6 (*CPA6,* rs1393902, *P* = 3.07 × 10^−7^) was a novel one. It is perhaps not surprising that the inclusion of two additional groups with differing ethnic backgrounds (Silk Road and TwinsUK) might change the overall significance of associations previously described in a more homogeneous collection of Southern European samples. The different results may simply indicate that the populations studied had differing contributions of genome variants to their hearing ability.

There are a number of limitations to this study. The threshold of significance used here (*P* ≤ 5 × 10^−8^) does not take into account the use of PCs providing three phenotypes (PC1, PC2 and PC3) (50). After a strictly conservative adjustment by Bonferroni, a significance threshold of *P* ≤ 1.7 × 10^−8^ would have been required to claim genome-wide significance, which was not reached in this study. However, despite being statistically independent phenotypes, all three PCs were derived from the same single measure of hearing ability.

Replication of the effect of *SIK3* rs681524 genotypes on hearing function would be useful. The samples included in this meta-analysis were of different ethnic populations, which increases the risk of population stratification. To adjust for this, population sub-structure was controlled for in the GWAS performed for each sample separately. Different ethnic backgrounds should also be considered when determining LD patterns of meta-analysis results. The LD pattern displayed in the locus zoom plot was based on LD as observed in the HapMap CEU population; however, this might not represent the LD pattern in some of the isolated populations studied here. We compared allele frequencies for the respective SNP in data from the Human Genome Diversity project (22) and found no significant differences in allele frequencies at rs681524 for the population presented here. The age range for the cohorts was very broad (18–98 years), and it did vary between the study groups, with the mean age of the samples (41.6–61.1) varying by 20 years. However, PCs were adjusted for age at hearing test prior to the GWAS. In addition, an association analysis stratified by age confirmed a consistent effect of the rs681524 across the age ranges that were largest in those under 40 years (Table 7). Although genotyping, imputation and GWA were performed for each cohort separately (Supplementary Material, Table S1), all analyses were conducted according to predefined protocols, to allow the use of the association results in meta-analysis and the direction of effect adjusted if necessary owing to differences in direction of PC loadings. Previous studies reported a gender difference in hearing ability particularly for older individuals (40). To control for possible gender differences in hearing ability, we adjusted for this in the GWAS of the mixed gender cohorts (all but TwinsUK). Furthermore, PCs rather than standard measures of hearing ability (i.e. pure-tone averages) were used to capture hearing ability. PCs might be more complex in their interpretation in respect to hearing, but they enable representation of both overall threshold shift and slope of the audiogram. In addition, PCs have been applied to capture pure-tone audiograms previously (14,17).

In conclusion, this study reports the findings of the largest meta-analysis of hearing function to date. The results suggest that *SIK3* is significantly associated with hearing ability in humans and expression work in mice supports a role for the expressed protein in cochlear function.

## MATERIALS AND METHODS

### Subjects

All samples included in this analysis were from the G-EAR consortium or TwinsUK (www.twinsuk.ac.uk). Both collections have been described in depth elsewhere (17,41). While samples from the G-EAR consortium were recruited from several isolated villages, TwinsUK samples were recruited as same-sex twin pairs of >18 years of age and resident in the UK. The specific TwinsUK sample presented here represents a subsample recruited to study ageing traits in females. Subjects from the Silk Road originate from five countries situated along the Silk Road (Azerbijan, Giorgia, Kazakistan, Tajikistan, Uzbekistan), which were combined as one cohort. Participants underwent a standard pure-tone audiogram according to recommendations by the local authorities (e.g. British Society of Audiology (42) for TwinsUK) and gave blood or saliva samples for DNA extraction. Participants with a reported family history of HL or previous ear diseases causing conductive HL were excluded from the analysis. All samples were recruited to represent hearing function in the population. All research was conducted according to the ethical standards as defined by the Helsinki declaration. The study was approved by the Institutional Review Board of IRCCS-Burlo Garofolo, Trieste, Italy and the National Research Ethics service London-Westminster (REC reference number: 07/H0802/84) as well as by the other involved members. Fully informed written consent was obtained from participants prior to their inclusion in the study.

### Phenotypes

Principal components were calculated from pure-tone thresholds measured in decibel hearing level (dB HL) (250, 500, 1 kHz, 2, 4 and 8 kHz) as previously described (14). While PCs for samples from TwinsUK, Korcula and Split were based on hearing thresholds for the better ear, PCA was conducted for the left and right ear separately in the remaining samples and the PC value for better ear selected afterwards. Eigenvector loadings for the different frequencies and PC eigenvalues for the different samples can be found in Supplementary Material, Table S3. The PCs represented the overall horizontal threshold shift, high-frequency slope and concavity of the audiogram, respectively, as described elsewhere (14). The size of PC1 gives information about the overall change in pure-tone thresholds, whereas PC2 is more sensitive to the slope in the audiogram, as seen for high-frequency sensorineural HL. PC1 is positively correlated with good hearing. Therefore, a high PC1 value represents overall low pure-tone thresholds (good hearing ability), whereas a low PC1 value indicates raised pure-tone thresholds over all frequencies (HL). PC2 was positively correlated with a high-frequency HL, with a high PC2 value representing a sloping audiogram and a low PC2 value indicating a flat, non-sloping audiogram. These conclusions were obtained by comparison of audiogram shapes between subjects with high, medium and low PC values for each PC1–3. Principal components were calculated separately for each cohort and adjusted for age and gender using linear regression. Age- and gender-adjusted residuals for PC1, 2 and 3 were rank-transformed to normality and used as traits for hearing ability. Direction of effect was adjusted prior to meta-analysis by taking the reverse of the effect if the direction of the PC loadings were of opposite direction between samples.

### Genotyping

Participants from the G-EAR consortium were genotyped on the Illumina 370 k chip or on the Affymetrix 500 k genotyping array. Genotyping and imputation were performed separately for each population. Subjects from the TwinsUK cohort were genotyped on either Illumina HumanHap300 Bead Chip or Illumina HumanHap610 Quad Chip (41). Genotype calling was performed using the appropriate software and imputed to the HapMap Phase 2 CEU sample. Genotyped SNPs were excluded from further analysis based on minor allele frequency (MAF), call rate and significance of violation from Hardy–Weinberg equilibrium (pHWE). Exact quality control measures applied for each population are shown in Supplementary Material, Table S1.

### Imputation

Imputation was performed separately for each of the eight populations based on haplotypes of the CEU Hapmap Phase II reference population. Populations from the G-EAR cohort (Italy, Croatia and Silk Road) were imputed using MACH 1.0 (Markov Chain-based haplotyper) (43,44), for TwinsUK using Impute version 2 (45). Imputed SNPs with a quality score of <0.4 (info in Impute versus 2) or 0.3 (Rsq in MACH 1.0), respectively, were excluded from the meta-analysis.

### Meta-analysis/statistical analysis

GWAS was run by each group using age- and gender-adjusted rank-transformed PC residuals. In the GWAS, a linear mixed-model regression was performed, assuming an additive genetic model. Owing to the increased relatedness of samples in the isolated populations and the twin sample presented here, relatedness was accounted for by inclusion of a kinship matrix in the polygenic model. This was performed using the GRAMMAR option (46) available for mmscore in Genabel (47). A comparison of three samples of related subjects has shown this option to be similar in power and type 1 error to using Genomic Control (48). In addition, for TwinsUK, population outliers (as determined by ancestry PCs) were excluded prior to analysis. Association analysis was performed for each of the eight samples separately.

The meta-analysis was performed by two centres independently using METAL (49), based on the weighted Z-score option. This method calculates a signed Z-score dependent on the *P*-value and direction of effect per allele per population. Z-scores for each allele and sample were weighted according to the sample size and summed to give a combined score. Extreme Z-scores (positive or negative) indicate a small *P*-value, whereas a positive score indicates increased, and a negative score a decreased disease risk (49). Meta-analysis was performed for PC1–3. The direction of effect (beta) for each sample and corresponding standard errors of the top associated genetic markers with PC2 are presented in plot (Fig. 2, with corresponding data in Table 5). An MAF threshold of ≥ 0.01, call rate of ≥0.90 and p(HWE) of ≥10^−6^ was applied in the meta-analysis. The combined betas were calculated as a summary measure of the separate population betas weighted by sample size of each population. LD information 500 kb up-and down-stream of rs681524 was extracted from the SNP Annotation and Proxy Search tool (24) using the 1000 Genomes pilot 1, CEU population dataset (*n* = 1092) (23) and the TwinsUK sample (*n* = 5654) newly imputed based on the whole-genome sequencing data from the UK10K TwinsUK cohort sample (*n* = 2000).

In addition to the TwinsUK sample, two further samples (Carlantino and Friuli Venezia Giulia) had whole genotyping data available in a subset (*n* = 93 and 222, respectively) (Table 6) and imputation accuracy was assessed in these populations. For TwinsUK, genotyping data from the original GWAS sample were compared with whole-genome sequencing data from the TwinsUK UK10K sample (Table 6).

### Analysis by age groups

The effect of the C allele at rs681524 on hearing was analysed in three age groups (>40, 40–60 and >60 years) in all samples separately. The association analyses were conducted adjusted for relatedness between subjects, as described earlier. PC values used in this analysis were not adjusted for age of subjects. A mean effect was calculated for all samples per age group (Table 7).

### Immunohistochemistry for Sik3

Mouse studies were carried out in accordance with UK Home Office regulations and the UK Animals (Scientific Procedures) Act of 1986 (ASPA) under a UK Home Office license, and the study was approved by the Wellcome Trust Sanger Institute's Ethical Review Committee. Mice were culled using methods approved under this license to minimize any possibility of suffering.

Wild-type C57Bl/6 mice homozygous for a spontaneous albino mutation were sacrificed at three different steps of development, the day of birth (P0), postnatal day 5 (P5) and at the age of 4 weeks. Mouse heads were fixed in 10% formalin at 4°C for 48 h, followed by two washing steps of 30 min in phosphate-buffered saline (PBS). For decalcification, 4-week-old specimens were kept in 10% ethylenediaminetetraacetic acid in PBS for 2.5 days at 4°C. Following the decalcification, tissues were washed in PBS (2 × 30 min at room temperature) and saline (2 × 2 h at 4°C). To dehydrate the samples, a dehydration chain of increasing ethanol concentration was used at 4°C. Tissues were embedded overnight and mounted in paraffin wax the following morning. Serial 8-µm sections were mounted on glass slides and dried at 40°C overnight. Immunohistochemistry was performed by the Ventana Discovery system (Ventana Medical Systems, Inc. Illkirch, France) according to the manufacturer's instructions.

For each age, between three and six mice and multiple sections per animal were analysed and observations only reported if all samples showed similar labelling patterns. Two separate Sik3 antibodies were used, and both gave identical labelling, supporting the validity of the expression patterns detected. Control sections were prepared omitting the primary antibody, and these showed no noticeable labelling.

### Antibodies

The following primary antibodies were used for immunohistochemistry experiments: primary Anti-SIK3 antibody (ab110987, Abcam, Cambridge, UK, and as a control: LS-c120369 SIK3 Lifespan Bioscience, Seattle, USA) at 1 : 50 (P0, P5) or 1 : 40 (4 weeks) concentration; anti-peripherin antibody (ab4666, Abcam, Cambridge, UK) at a 1 : 100 concentration; anti-TUJ1 antibody (Covance, New Jersey, US) at a 1 : 100 concentration; anti-GFAP antibody (ab53554, Abcam, Cambridge, UK) at a 1 : 50 concentration. The secondary antibody, biotin-conjugated donkey anti-rabbit (711-065-152), was purchased from Jackson ImmunoResearch (West Grove, PA, USA). Images of the antibody stained sections were taken using a Zeiss Axioskop MOT light microscope. Image processing was performed in Adobe Photoshop CS5.

### Confocal microscopy

Heads from 5-day-old mice (P5) were bisected, and inner ears plus bone were removed from the skull and then fixed in 4% paraformaldehyde for 2 h at room temperature. Subsequently, specimens were fine-dissected in PBS, then washed and permeabilized in 1% PBS/Triton X-100 (PBT) and blocked with 10% sheep serum. Then, they were incubated with the primary antibody, rabbit polyclonal against Sik3 (ab110987, Abcam, Cambridge, UK, dilution 1 : 200) overnight at 4°C. After washes with PBT, samples were incubated with anti-rabbit Alexa Fluor 488 secondary antibody (Invitrogen, anti-rabbit, diluted 1 : 500) and rhodamine/phalloidin (Invitrogen, diluted 1 : 100). Samples were mounted in Prolong Gold Antifading reagent (Invitrogen). Images were acquired on a LSM 510 Meta confocal microscope (Zeiss, Welwyn Garden City). Image processing was performed in Adobe Photoshop CS5.

## SUPPLEMENTARY MATERIAL

Supplementary Material is available at *HMG* online.

## FUNDING

This work was supported by a joint PhD studentship from Action on Hearing Loss and AgeUK. TwinsUK—the study was funded by the Wellcome Trust; European Community′s Seventh Framework Programme (FP7/2007-2013). The study also receives support from the National Institute for Health Research (NIHR) BioResource Clinical Research Facility and Biomedical Research Centre based at Guy's and St Thomas’ NHS Foundation Trust and King's College London. This work was further supported by the Wellcome Trust [grant numbers 098051 and 100669] and thanks to funds from Ministry of Health (Ricerca Finalizzata and Ricerca Corrente), Fondo Trieste and Parco Genetico Grant from FVG Regonal Government to P.G. The Cilento study was supported by Fondazione con il SUD [2011-PDR-13] and Ministry of Education, Universities and Research (INTEROMICS Flag Project) to M.C. The CROATIA-Split and CROATIA-Korcula studies were funded by grants from the Medical Research Council (UK), European Commission Framework 6 project EUROSPAN (Contract No. LSHG-CT-2006-018947) and Republic of Croatia Ministry of Science, Education and Sports research grants to I.R. [108-1080315-0302]. Maria Pina Concas was supported by the Italian Ministry of Education, University and Research (MIUR) grant no. 5571/DSPAR/2002. The funders had no role in study design, data collection and analysis, decision to publish, or preparation of the manuscript. Funding to pay the Open Access publication charges for this article was provided by the Wellcome Trust.

## Supplementary Material

Supplementary Data
