# Supplementary material for: Salt-inducible kinase 3, SIK3, is a new gene associated with hearing
Source: Hum Mol Genet. 2014 Jul 24;23(23):6407–18. doi: 10.1093/hmg/ddu346 (PMC4222365; doi:10.1093/hmg/ddu346)
Supplement: Supplementary Data [file supp_23_23_6407__index.html]

Salt-inducible kinase 3, SIK3, is a new gene associated with hearing — Salt-inducible kinase 3, SIK3, is a new gene associated with hearing — Salt-inducible kinase 3, SIK3, is a new gene associated with hearing — Supplementary Data 

# Salt-inducible kinase 3, *SIK3*, is a new gene associated with hearing

## Supplementary Data

Supplementary Data

**Files in this Data Supplement:**

- Supplementary Data - Supplementary Data
- Supplementary Data - Supplementary Data
- Supplementary Data - Supplementary Data
