# Supplementary material for: Salt-inducible kinase 3, SIK3, is a new gene associated with hearing
Source: Hum Mol Genet. 2014 Jul 24;23(23):6407–18. doi: 10.1093/hmg/ddu346 (PMC4222365; doi:10.1093/hmg/ddu346)
Supplement: Supplementary Data [file supp_ddu346_ddu346supp3.pdf]

**CILENTO**	PC1	PC2	PC3
Eigenvalue	3.267	1.147	0.684
Eigenvector loadings for the right ear:			
X250_DX	-0.1736698	-0.2539901	0.49104283
X500_DX	-0.2659465	-0.3233838	0.47595626
X1K_DX	-0.3539639	-0.351799	0.16881825
X2K_DX	-0.5286937	-0.2693573	-0.296288
X4K_DX	-0.5358539	0.0666892	-0.5285676
X8K_DX	-0.4551532	0.79381898	0.36969273
	PC1	PC2	PC3
Eigenvalue	3.182	1.224	0.696
Eigenvector loadings for the left ear:			
X250_SN	-0.1858338	0.44759875	-0.2883511
X500_SN	-0.24123	0.52104835	-0.2796163
X1K_SN	-0.3461424	0.45851868	0.08354126
X2K_SN	-0.5806534	0.00405049	0.46794228
X4K_SN	-0.5471331	-0.3809958	0.20767688
X8K_SN	-0.3885181	-0.4156325	-0.7547116

**SILK ROAD**	PC1	PC2	PC3
Eigenvalue	3.1035029	1.0222101	0.6427502
Eigenvector loadings for the right ear:			
X250_DX	-0.2933278	-0.2280827	-0.1479005
X500_DX	-0.2959039	-0.2616893	-0.2331844
X1K_DX	-0.3619184	-0.2863678	-0.1666671
X2K_DX	-0.5513963	-0.3237385	-0.0923952
X4K_DX	-0.4898067	0.23674149	0.82210018
X8K_DX	-0.3891867	0.79789555	-0.4599858
	PC1	PC2	PC3
Eigenvalue	2.7954637	1.203024	0.7147479
Eigenvector loadings for the left ear:			
X250_SN	-0.2051011	-0.2439315	-0.3457846
X500_SN	-0.1995683	-0.2276802	-0.2391568
X1K_SN	-0.2983308	-0.3383372	-0.2876289
X2K_SN	-0.6095508	-0.448492	0.18262183
X4K_SN	-0.5501183	0.3895796	0.59470033
X8K_SN	-0.393602	0.64905299	-0.5945481

FRUILI VENEZIA GUILIA	PC1	PC2	PC3
Eigenvalue	3.6019159	1.0768151	0.4941869
Eigenvector loadings for the right ear:			
X250_DX	-0.3528555	-0.372105	-0.1613832
X500_DX	-0.3688955	-0.4101265	-0.1674097
X1K_DX	-0.3740096	-0.3585938	-0.0210505
X2K_DX	-0.4211818	-0.1328451	0.25243221
X4K_DX	-0.4564531	0.41600173	0.68978207
X8K_DX	-0.4623661	0.61158753	-0.6371539
	PC1	PC2	PC3
Eigenvalue	3.7792568	1.050853	0.4459372
Eigenevector loadings for the left ear:			
X250_SN	-0.3159907	-0.3674399	-0.2076766
X500_SN	-0.3537587	-0.4079889	-0.1692703
X1K_SN	-0.3910425	-0.3758088	-0.0315102
X2K_SN	-0.4354739	-0.1488115	0.28701067
X4K_SN	-0.4598637	0.40215346	0.67055167
X8K_SN	-0.4700833	0.61108788	-0.6286577

CARLANTINO	PC1	PC2	PC3
Eigenvalue	3.4884639	0.8817346	0.6300651
Eigenvector loadings for the right ear:			
X250_DX	-0.2381001	0.20325608	0.24993696
X500_DX	-0.3877411	0.36473847	0.42308564
X1K_DX	-0.4590671	0.34873256	0.10081004
X2K_DX	-0.5169611	0.20461555	-0.6289698
X4K_DX	-0.4463747	-0.6131471	-0.2766257
X8K_DX	-0.3401815	-0.5349967	0.52558793
	PC1	PC2	PC3
Eigenvalue	3.376992	0.8882479	0.6796592
Eienvector loadings for the left ear:			
X250_SN	-0.2104544	-0.1135853	0.28105826
X500_SN	-0.3449249	-0.2575987	0.50387198
X1K_SN	-0.4476488	-0.3061557	0.1267186
X2K_SN	-0.5310599	-0.4277243	-0.4915955
X4K_SN	-0.4376513	0.55857517	-0.4677973
X8K_SN	-0.4034638	0.57624274	0.4365332

**TALANA**	PC1	PC2	PC3
Eigenvalue	1.6547434	1.3640165	1.1228096
Eigenvector loadings for the right ear:			
X250_DX	-0.4764095	-0.3186264	0.33614678
X500_DX	-0.3327436	-0.26272	0.59557482
X1K_DX	-0.2782142	0.63298885	0.10269807
X2K_DX	-0.1989331	0.64753078	0.24609485
X4K_DX	-0.5990861	-0.0953321	-0.2823851
X8K_DX	-0.4317801	-0.019905	-0.6176124
	PC1	PC2	PC3
Eigenvalue	2.1434644	1.4479261	0.9990374
Eigenvector loadings for the left ear:			
X250_SN	-0.5166048	0.48322921	-0.0665158
X500_SN	-0.5338121	0.45635177	-0.0364329
X1K_SN	-0.4645173	-0.4893464	-0.1243889
X2K_SN	-0.4357014	-0.5411629	-0.1354123
X4K_SN	0.10869081	0.01965556	-0.882693
X8K_SN	-0.175324	-0.1597779	0.42578439

TwinsUK	PC1	PC2	PC3
Eigenvalue	3.549	1.223	0.5122
Eigenvector loadings for the better ear:			
X250	0.352	0.549	0.428
X500	0.41	0.476	0.073
X1000	0.455	0.141	-0.506
X2000	0.441	-0.25	-0.476
X4000	0.417	-0.408	0.134
X8000	0.365	-0.472	0.557

**Split**	PC1	PC2	PC3
Eigenvalue	3.108	1.116	0.555
Eigenvector loadings for the better ear:			
X250			
X500			
X1000			
X2000			
X4000			
X8000			

**Korcula**	PC1	PC2	PC3
Eigenvalue	3.212	1.210	0.493
Eigenvector loadings for the better ear:			
X250			
X500			
X1000			
X2000			
X4000			
X8000			
