# Supplementary material for: Salt-inducible kinase 3, SIK3, is a new gene associated with hearing
Source: Hum Mol Genet. 2014 Jul 24;23(23):6407–18. doi: 10.1093/hmg/ddu346 (PMC4222365; doi:10.1093/hmg/ddu346)
Supplement: Supplementary Data [file supp_ddu346_ddu346supp1.docx]

| **Population** | **array** | **genotype calling** | **QC criteria** | **imputation** | **imputation reference population** | **statistical analysis tool** |
| --- | --- | --- | --- | --- | --- | --- |
| Carlantino | Illumina 370 CNV | Bead studio | call rate>90%  p(hwe)>10^-4^ | MACH | HapMap Phase II | GenABEL, ProbABEL |
| Friuli Venezia Guilia | Illumina 370 CNV | Bead studio | call rate>90%  p(hwe)>10^-4^ | MACH | HapMap Phase II | GenABEL, ProbABEL |
| Korcula | Illumina 370 CNV | GenomeStudio | call rate>98%  p(hwe)>10^-10^ | MACH | HapMap Phase II | GenABEL, ProbABEL |
| Split | Illumina 370 CNV | GenomeStudio | call rate>98%  p(hwe)>10^-10^ | MACH | HapMap Phase II | GenABEL, ProbABEL |
| Cilento | Illumina 370 K | Illumina | call rate>95%  SNPs not in hapmap | MACH | HapMap Phase II | GenABEL, ProbABEL |
| Talana | Affymetrix 500 K | BRLMM | pHWE<1e-6, call rate 95%, MAF 0.01 | MACH | HapMap Phase II | GenABEL, ProbABEL |
| Silk Road | Illumina 700 K | GenomeStudio | call rate>97%  p(hwe)>10^-8^ | MACH | HapMap Phase II | GenABEL, ProbABEL |
| Twins UK | Illumina HumanHap300 Bead Chip  Illumina HumanHap610 Quad Chip | Illuminus algorithm | call rate≥97% (SNPs with MAF≥5%)  call rate≥99% (for 1% ≤ MAF< 5%)  p(hwe)>10^-6^  MAF≥ 1% | Impute vs2 | HapMap Phase II | GenABEL |

**S1 Genotyping and imputation information per population**

S1 Genotyping and imputation information per population

The number of genotyped SNPs taken forward for imputation was based on passing the defined quality control (QC) criteria (p(hwe)= significance of deviation from Hardy Weinberg equilibrium, MAF=minor allele frequency, call rate=genotyping efficiency per SNP). Imputation was performed in Markov Chain based haplotyper (MACH) or Impute vs2 based on the HapMap CEU based on haplotypes of the HapMap Phase II reference population. As statistical tools for genome-wide association analyses ProbABLE and GenABEL in R were used.
