# Supplementary figures and images for: Salt-inducible kinase 3, SIK3, is a new gene associated with hearing
Source: Hum Mol Genet. 2014 Jul 24;23(23):6407–18. doi: 10.1093/hmg/ddu346 (PMC4222365; doi:10.1093/hmg/ddu346)

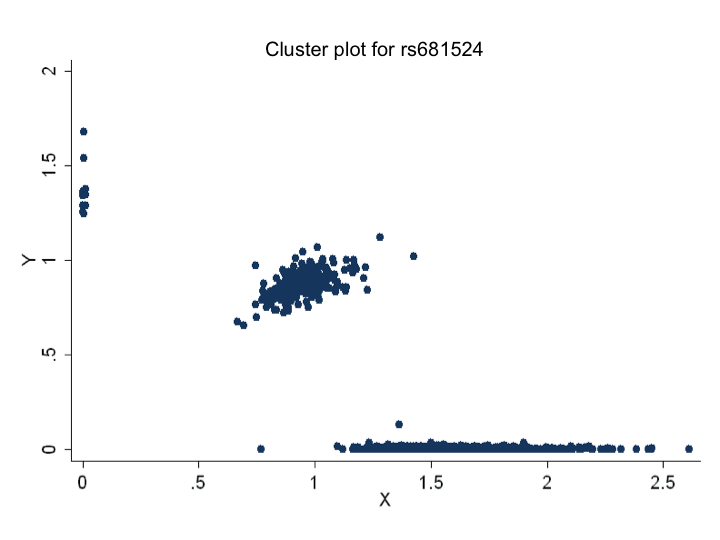

Supplement: Supplementary Data [file supp_ddu346_ddu346supp2.png]
